# Unique Neural Activity Patterns Among Lower Order Cortices and Shared Patterns Among Higher Order Cortices During Processing of Similar Shapes With Different Stimulus Types

**DOI:** 10.1177/20416695211018222

**Published:** 2021-05-26

**Authors:** Zhen Li, Hiroaki Shigemasu

**Affiliations:** Department of Psychology, The University of Hong Kong, Hong Kong, China; Graduate School of Engineering, 47743 Kochi University of Technology, Kochi, Japan; School of Information, 47743 Kochi University of Technology, Kochi, Japan

**Keywords:** shape representation, depth cues, functional magnetic resonance imaging, multivoxel pattern analysis

## Abstract

We investigated the neural mechanism of the processing of three-dimensional (3D) shapes defined by disparity and perspective. We measured blood oxygenation level-dependent signals as participants viewed and classified 3D images of convex–concave shapes. According to the cue (disparity or perspective) and element type (random dots or black and white dotted lines), three types of stimuli were used: random dot stereogram, black and white dotted lines with perspective, and black and white dotted lines with binocular disparity. The blood oxygenation level-dependent images were then classified by multivoxel pattern analysis. To identify areas selective to shape, we assessed convex–concave classification accuracy with classifiers trained and tested using signals evoked by the same stimulus type (same cue and element type). To identify cortical regions with similar neural activity patterns regardless of stimulus type, we assessed the convex–concave classification accuracy of transfer classification in which classifiers were trained and tested using different stimulus types (different cues or element types). Classification accuracy using the same stimulus type was high in the early visual areas and subregions of the intraparietal sulcus (IPS), whereas transfer classification accuracy was high in the dorsal subregions of the IPS. These results indicate that the early visual areas process the specific features of stimuli, whereas the IPS regions perform more generalized processing of 3D shapes, independent of a specific stimulus type.

## Introduction

The ability to perceive the visual world in three dimensions is critical for survival. In humans, most activities of daily life depend on the ability to manipulate objects and the self in a three-dimensional (3D) space. However, the images projected onto the retina are two-dimensional and so must be reconstructed into an accurate 3D representation by the visual system. The information (cues) used for 3D reconstructions are termed *depth cues*. Depth cues can be classified into two types: binocular cues that depend on the difference in visual information acquired by the two retinas (binocular disparities) and monocular cues that can be acquired by only one eye and include perspective, texture, motion parallax, retinal image size, and interposition.

This 3D reconstruction involves a series of processing steps from the retina to higher order visual cortices. According to the two-stream theory, visual information is processed progressively through the retina, thalamus, and primary visual cortex (V1) in the occipital lobe and then divided into two anatomically and functionally separate streams—a dorsal stream from V1 to the parietal cortex and a ventral stream from V1 to the temporal cortex. Each stream processes visual information in a hierarchical manner, with each cortical region processing information based on the results (output) of lower order regions (Zeki, 1978). Meanwhile, visual information processing is not strictly hierarchical: there is feedback from higher to lower areas and lateral interactions within areas (Lamme et al., 1998; Ramalingam et al., 2013). Mishkin and Ungerleider (1982) first conceptualized the two-stream theory based on lesion research in non-human primates. The ventral stream has been termed the “what” stream as it processes information related to an object’s identity (e.g., shape, size, color, texture), whereas the dorsal stream has been termed the “where” stream as it processes visual information related to an object’s location, movement, and spatial relationships. According to this perspective, 3D shape information should be processed by the ventral stream.

 A revised two-stream theory was formulated by Goodale and Milner (1992). Rather than viewing both streams as contributing to conscious visual awareness, they argued that only the ventral stream contributes to conscious vision (known as the “perception” stream), while information in the dorsal pathway is used for the unconscious control of action, such as the movement of the body guided by visual input (and is therefore known as the “action” stream). According to this view, 3D shape information can also be processed in the dorsal stream for visually guided action. In fact, there is evidence that 3D shape information is processed in both the dorsal and the ventral streams. In a macaque monkey study, Janssen et al. (1999) found that neurons in a subregion of the inferior temporal cortex in the ventral stream were selective to 3D shape defined by disparity, while a human functional magnetic resonance imaging (fMRI) study by Taira et al. (2001) found that the intraparietal area in the dorsal stream contributes to the perception of 3D surface structure based on shading. Furthermore, an fMRI study by Freud et al. (2017) of patients with visual object agnosia due to ventral cortex injury revealed that the intact dorsal cortex can produce 3D object representations without input from the ventral stream.

Studies have been conducted to discover how 3D information is processed from a single cue, such as disparity (Orban et al., 2006) and pictorial cues (Cauquil et al., 2005; Todd, 2010). There have also been studies that have investigated 3D shape perception when different cues work together (Dövencioğlu et al., 2013; Murphy et al., 2013; Saunders & Chen, 2015; Yamane et al., 2008). However, the understanding of 3D shape processing is still not complete. The first aim of this study is to investigate which visual areas are responsive to the specific 3D shape used. To this end, we measured the blood oxygenation level-dependent (BOLD) signal in visual pathways using fMRI (Logothetis et al., 2001) while subjects viewed 3D images of two simple shapes (convex or concave) composed of different visual elements and with distinct depth cues. Then, multivoxel pattern analysis (MVPA) was performed to classify the shapes using the BOLD signal patterns in various regions of interest (ROIs), including the retinotopic visual cortices (V1, V2, V3d, V3v, V3A, and V7), the higher ventral cortex (lateral occipital complex [LOC]), the higher dorsal area (the human middle temporal complex [hMT+] and kinetic occipital area [KO]), and the intraparietal sulcus (IPS) areas (the ventral intraparietal sulcus [VIPS]; the parieto-occipital intraparietal sulcus [POIPS]; and the dorsal intraparietal sulcus [DIPS]). The classifier was trained and tested using activity patterns in response to the images (convex or concave) with the same type of stimuli (same depth cue and visual element), a condition which is termed “same-type stimuli convex versus concave classification.” If the classification accuracy using the signal from a given ROI is higher than the statistical significance level, we can conclude that the neural activity pattern in the ROI includes information, which correlates to the 3D shapes from a specific type of stimuli, and this information may be used to differentiate 3D shapes from a specific type of stimuli by the human visual system; however, we are unable to test whether it is actually used in this study (Schalk et al., 2017; Williams et al., 2007).

A variety of depth cues contribute to 3D shape perception, and many models have been proposed to explain how different depth cues contribute to 3D shape processing at the neural level. Maloney and Landy (1989) proposed a simple statistical framework for combining depth estimates from consistent depth cues in which information from different cues is processed independently by different modules and then fused into a single depth estimate about each point of the scene. They also proposed that the assigned weights of different estimates are variable and that the combination is linear (additive). In contrast, Clark and Yuille (1990) distinguished between “strong fusion” and “weak fusion.” In strong fusion, information from different cues interacts and is processed cooperatively to yield a single depth estimate, whereas in weak fusion, information from different cues is independent and the final estimate is obtained by combining the individual information. Regarding the functions of specific cortical structures in the processing of different depth cues and their potential interactions, there are two broad possibilities: (a) regional neural activity patterns differ depending on cue type(s) or (b) shape information from different depth cues is fused so that neural activity patterns are similar among some regions, irrespective of cue type. Some researchers have investigated questions related to these possibilities. For example, Ban et al. (2012) found that human dorsal area V3B/KO is involved in the integration of motion and disparity cues by fMRI study. More recently, using the same technique, Armendariz et al. (2019) provided evidence that the macaque middle temporal (MT) area computes the fusion of disparity and motion depth cues, which is similar to the role of the human V3B/KO. Tsutsui et al. (2001) found that the caudal part of the lateral bank of the intraparietal sulcus (area caudal intraparietal region [CIP]) may be involved in the integration of perspective and binocular cues for the perception of surface orientation in depth. Similar to these studies, we investigated the neural patterns for our specific stimuli. The second aim of this study is to investigate whether the neural activity patterns differ during the processing of a given 3D shape with distinct depth cues/elements or if there are common patterns among some regions independent of cue types/elements. We examined this using MVPA to classify BOLD data for each ROI obtained during the processing of images with cues and element types that differed from that of the training set, a condition termed “transfer convex versus concave stimuli classification.” When the classification accuracy is higher than the statistical significance level in a given ROI, this suggests that there are common neural activity patterns for processing shapes using different types of stimuli (i.e., the activity pattern in a given ROI during processing of the same shape is independent of the depth cue or element type).

Early visual areas are believed to process simple attributes; for example, neurons in V1 are selective to orientation, binocular disparity, and motion direction (Hubel & Wiesel, 1962). For the same stimuli convex versus concave classification task, these local attributes of convex and concave shape are very different. Therefore, the first hypothesis is that some early areas show high classification accuracy for the “same-type stimuli convex versus concave classification.” For the higher level visual areas, there is evidence that some higher order dorsal and ventral stream regions are related to shape processing (Todd, 2004). Therefore, the second hypothesis is that these areas may show high classification accuracy for the “same-type stimuli convex versus concave classification.” Furthermore, these regions process more complex object attributes, and there is evidence that certain parietal areas are involved in processing 3D shapes defined by different cues. For example, an fMRI study by Durand et al. (2009) revealed that the anterior IPS regions are involved in the processing of 3D shapes defined by disparity, and Nelissen et al. (2009) found that the processing of 3D shapes based on texture involves both ventral and parietal regions, with the strongest activation observed in the CIP, with decreasing strength toward the anterior IPS (Nelissen et al., 2009). In addition, the dorsal visual stream is believed to be involved in the unconscious control of action. For instance, the intraparietal cortex is involved in the control of visually guided actions, such as reach-to-grasp, which require the rapid extraction of 3D shape information. The 3D shape information required to guide actions may be more generalized and does not depend on the specific nature of the depth cues and elements. Information processing in the dorsal stream generally follows the trend of the more anterior areas and are more likely to be concerned with computations related to potential action (Fabbri et al., 2016; Shmuelof & Zohary, 2005; Stark & Zohary, 2008). Therefore, it is possible that neurons in higher areas, especially the parietal areas, will show a common neural activity pattern when 3D shapes defined by different types of stimuli are presented; therefore, we also hypothesized that higher areas (especially parietal areas) would show high accuracy for “transfer convex versus concave classification.”

## Methods

### Ethics Statement

The study protocol was approved by the Human Research Ethics Committee of the Kochi University of Technology. Written informed consent was obtained from all participants in accordance with the Declaration of Helsinki.

### Participants

Nine participants were recruited for the fMRI experiments (seven males and two females). One male was left-handed, and all other participants were right-handed. All participants had normal or corrected-to-normal vision. None of the patients had a history of mental illness or neurological disease. Their ages ranged from 20 to 34 years (mean 23.9 ± standard deviation 3.9 years). Participants were remunerated for their participation.

### Stimuli

Stereoscopic stimuli were presented using a PROPixx projector with a 3D circular polarized filter placed in front. The stimuli were projected onto a translucent screen inside the bore of the fMRI magnet. The participants wore polarized glasses and viewed the images through a tilted mirror (angled at 45°) located above the head coil. The optical distance from the midpoint of the two eyes to the screen was 71 cm. The screen resolution was set at 1,920 × 1,080 pixels and the refresh rate was 480 Hz.

**Figure 1.**
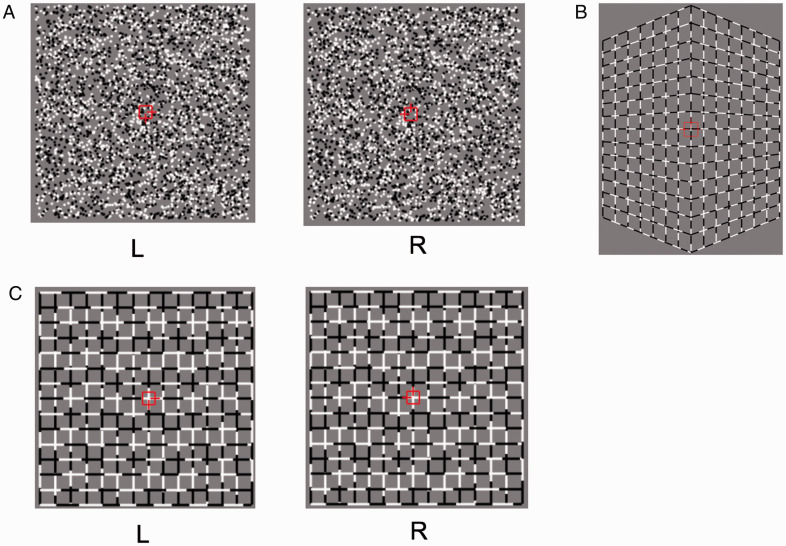
Sample visual stimuli used to generate 3D images of convex or concave shapes, which consisted of planes (only those generating the convex shape are shown). A: Random dot stereogram. B: Black and white dotted lines with perspective. C: Black and white dotted lines with disparity.

The 3D stimuli were generated using Psychotoolbox 3 in MATLAB (MathWorks, Natick, MA, USA). The images depicted a shape that was either convex or concave and consisted of two slanted planes. According to different visual components and depth cues, we used three types of stimuli: (A) random dot stereogram (RDS), (B) black and white dotted lines with perspective, or (C) black and white dotted lines with disparity (Figure 1). Different depth cues with similar elements (Stimulus Type B and Type C) were used to examine whether cues could induce cue-specific or common neural activity patterns. Similarly, images with different elements and the same depth cues (Stimulus Type A and Type C) were used to assess activity related to visual elements. Images were presented on a mid-gray background rectangular area of 31.7° × 18.2°. A fixation marker comprising a hollow square with a side length of 0.7° and horizontal and vertical nonius lines of 0.5° was shown at the center of the screen to help participants maintain eye vergence.

**Figure 2.**
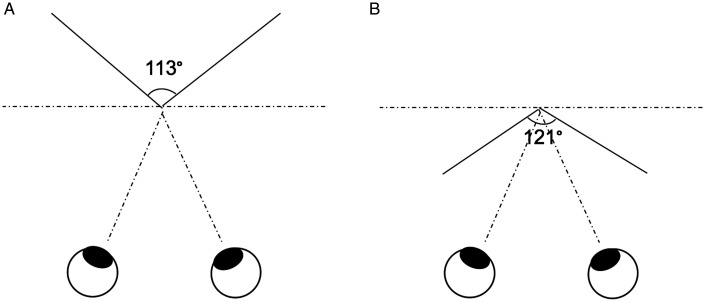
Top views of the virtual 3D images (using the RDS stimuli as the example). A: The convex shape. B: The concave shape.

For the RDS, the stimulus for each eye covered an area of 14.6° × 14.6°. The density of the stereogram was 289 dots/deg^2^, and all dots were the same size (0.2° in diameter). The relative disparity between the peak and the sides of the convex shape was 0.4° and that of the concave shape was 0.2°. The angle of the two slanted planes forming the convex shape was 113° and that of the planes forming the concave shape was 121°. Both the convex and the concave peaks were located at the fixation marker plane. The concave shape was nearer than the fixation marker plane with crossed disparity, whereas the convex shape was farther than the fixation marker plane with uncrossed disparity. Figure 2 shows the top view of the virtual 3D images.

For the stereo images composed of black–white dotted lines with disparity, each monocular component covered an area of 14.6° × 14.6°. Lines consisted of black and white sublines of random lengths. We used dotted lines because it is easier for participants to perceive convex and concave shapes compared with solid black or white lines with disparity. In the latter case, participants may not be able to correctly match the corresponding lines in the left and right eyes. The angle of the two slanted planes forming these convex and concave images differed among the participants. To determine the optimal angle for 3D perception, participants first performed a task to adjust the angle defined by the dotted lines with disparity to that defined by the RDS inside the bore of the scanner before the fMRI scan. In this procedure, the reference shape defined by the RDS and the shape for adjusting the dotted lines with disparity were shown in sequence, and the participants adjusted the angle of the latter image by pressing the corresponding buttons on a keypad. This task was repeated twice by each participant, and the average disparity was used for the main experiment.

For the images composed of black and white dotted lines with perspective, we used the same types of lines as those used for the images with disparity. The participants performed a similar angle adjustment task so that the angle of the shape defined by the perspective appeared the same as the angle of the shape defined by the RDS. The width of the stimuli was 14.6° and the height differed depending on the angle adjustment determined by the participants.

### Experimental Design

**Figure 3.**
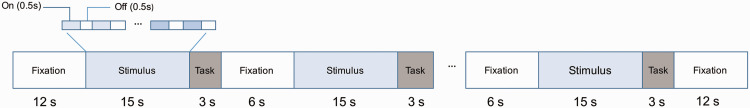
The block design for the image-classification task during functional magnetic resonance imaging.

Convex and concave shapes composed of different elements or cues were presented: (a) RDS, (b) black and white dotted lines with perspective, and (c) black and white dotted lines with disparity. Thus, there were six different stimuli (3 types × 2 shapes), which were presented in a block design (Figure 3). To avoid adaptation and to maintain neuronal activation, the stimuli in each block were flashed on and off at 1 Hz (with each on and off period lasting 0.5 seconds). The random dots or black and white dotted lines were regenerated on every presentation. In each block, one of the six stimulus conditions was shown for 15 seconds. After each block, the participant was required to judge whether the shape was convex or concave by pressing the corresponding button on a keyboard within 3 seconds. After each judgment, there was a 6 seconds fixation period (the last fixation block was 12 seconds). The six stimulus conditions were presented in random order (12 blocks). Each run began with a 12 seconds fixation period and a total run lasted 306 seconds.

The day before the fMRI scans, participants were asked to perform two tests in front of a computer screen outside the scanner to assess whether participants were qualified for our experiment: (a) we showed all the stimuli (i.e., the convex and concave shapes used in fMRI scan) individually and asked the participants to report the shape they observed, to which the participants responded verbally. The participants that successfully reported all the shapes were eligible for the second test. (b) Convex versus concave discrimination test, similar to the one used during fMRI scan, was performed. The participants first practiced the test for two runs, after which they performed the formal test for maximum three runs. The participants with all answers correct in one of the runs were then recruited for the formal fMRI experiment.

During the fMRI scan, participants were required to observe the fixation marker during the scan, and to try their best to reduce head movement as much as possible over the entire session. If head movement was too large during one run (translational movement > 2 mm or rotation > 2°), the run was excluded from the analysis. In addition, if two or more behavioral errors occurred of any stimulus type in one run, this run was excluded from the analysis. In general, all participants were able to maintain head stillness and performed well, and at least six runs of echo-planar imaging (EPI) data were acquired for analysis from each participant.

### fMRI Data Acquisition

Functional MRI data acquisition was performed at the Brain Communication Research Centre of the Kochi University of Technology using a 3-Tesla Siemens MRI scanner with a 24-channel multiphase array head coil. A pair of foam pads was used to help participants to keep their head still during the experiment. To construct a flattened cortical surface with preserved relative dimensions, a high resolution (1 × 1 × 1 mm) T1-weighted anatomical image was obtained for each participant. During the functional scans, we measured the BOLD signals using an EPI sequence from an area of 35 slices that covered the visual cortex, posterior parietal cortex, and posterior temporal cortex. The acquisition parameters of the EPI sequence were as follows: 102 volumes per run, echo time of 30 milliseconds, repetition time of 3,000 milliseconds, slice thickness of 3 mm, in-plane resolution of 2.04 mm × 2.04 mm, and descending slice acquisition order. For each session, we also measured a T2-weighted structural image with the same number of slices, which covered the same area as the corresponding EPI data. This structural image was used as the reference for 3D motion correction of EPI data and for coregistration between the T1-weighted anatomical image and the EPI images. After coregistration, both the anatomical image and the EPI images were transformed into Talairach space.

**Figure 4.**
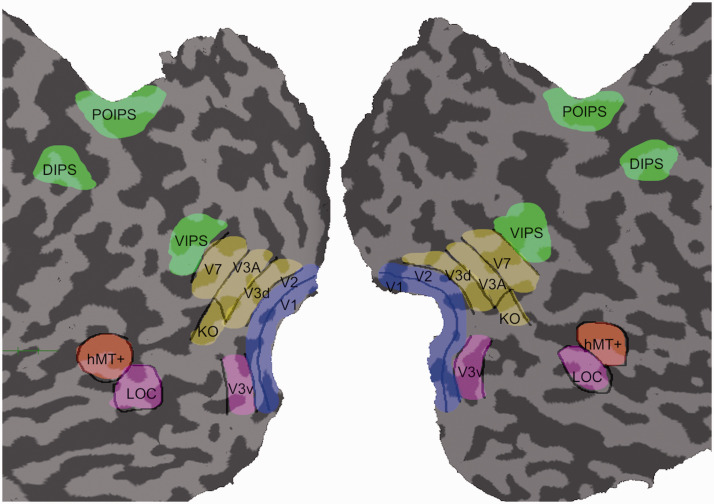
Illustration of regions of interests superimposed on the flattened visual cortex. LOC = lateral occipital complex; hMT+ = the human middle temporal complex; KO = kinetic occipital area; VIPS = ventral intraparietal sulcus; POIPS = parieto-occipital intraparietal sulcus; DIPS = dorsal intraparietal sulcus.

**Figure 5.**
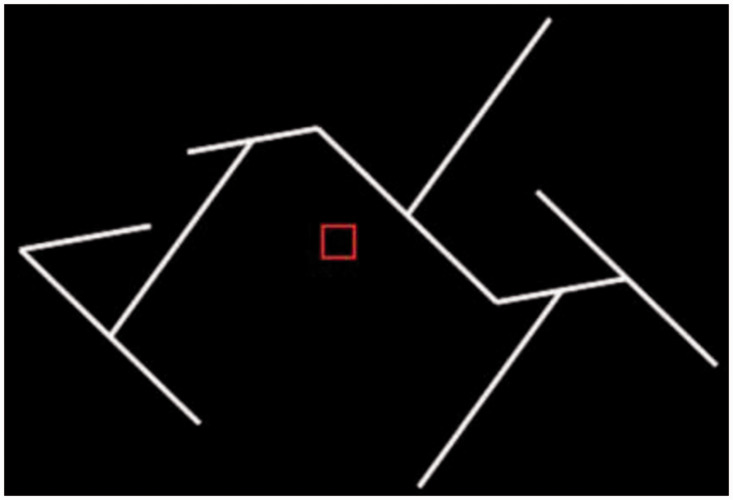
Illustration of IPS areas localizer.

To define the ROIs, separate fMRI measurements were acquired from all participants before the main experiment using stimuli shown to activate specific cortical regions in previous studies (Figure 4). A rotating wedge and expanding rings were used to define the retinotopically organized visual areas V1, V2, V3d, V3v, and V3A (DeYoe et al., 1996; Sereno et al., 1995; Warnking et al., 2002). The V7 was defined as the area dorsal and anterior to V3A with lower visual field quadrant representation (Tootell et al., 1998; Tyler et al., 2005). In addition, the higher dorsal areas hMT+ and KO, the higher ventral area LOC, and areas along the IPS (VIPS, POIPS, and DIPS) were localized using standard procedures. hMT+ was defined as an area of voxels in the lateral temporal cortex exhibiting higher activation to dots that moved outward and inward coherently compared with static dots (Zeki et al., 1991), while KO was defined as an area of voxels showing higher activation in response to contours defined by motion compared with the transparent motion of black and white dots (Dupont et al., 1997; Zeki et al., 2003). LOC was defined as an area of voxels in the lateral occipito-temporal cortex demonstrating higher activation in response to intact images of objects compared with the corresponding scrambled image (Kourtzi & Kanwisher, 2000, 2001). Finally, individual areas along the IPS (VIPS, POIPS, and DIPS) were localized using nine connected lines as stimuli (Figure 5). These areas were identified by contrasting activity to 3D shapes produced by rotating the lines in depth compared with movement along a frontoparallel plane (Vanduffel et al., 2002).

### Data Analysis 

#### Preprocessing

Data processing and analysis were conducted using Freesurfer (Fischl, 2012), BrainVoyager QX (Version 2.8.4.2645, 64-bit; BrainInnovation, Maastricht, the Netherlands), MATLAB R2014a, and SPSS Statistics 23 (IBM Inc.). The scalp was removed from the T1-weighted 3D anatomical images of each participant, and the white matter (WM) was separated from the other components using Freesurfer. The WM component was then used as the mask to segment WM from gray matter (GM) using Brain Voyager QX. The extracted brain was transformed into Talairach space using BrainVoyager QX. The flattened cortical surface was generated to visualize the functional maps and to delineate the ROIs by the following series of operations: segmenting the brain along the GM/WM boundary, inflating the segmented GM, cutting the GM along the calcarine sulcus, and flattening. The ROIs were then used for MVPA. For EPI data, slice scan time correction was performed. Then, 3D motion correction was performed using a T2-weighted reference image obtained at the beginning of each session (Woods et al., 1998). Temporal filtering was then applied on these EPI data. Spatial smoothing was not performed. For coregistration, T2-weighted data and T1-weighted data were used to estimate the parameters. After that, these parameters were applied to the EPI data; therefore, EPI data and T1-weighted data were coregistered. Finally, functional EPI data were transformed into Talairach space.

#### ROI-Based MVPA

**Figure 6.**
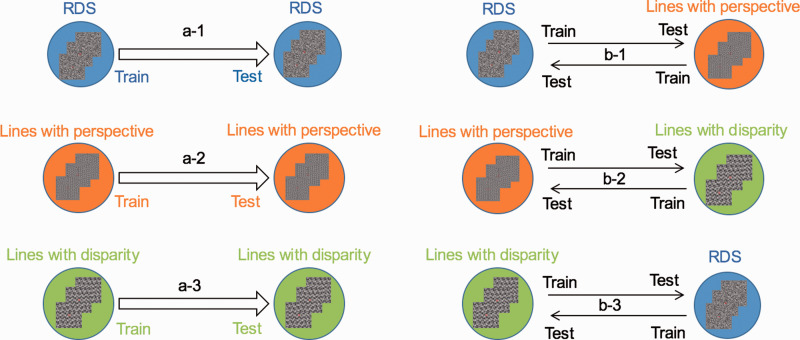
Illustration of the classifications performed. a-1, a-2, and a-3 indicate classification of convex versus concave 3D images generated with the same type of stimuli; b-1, b-2, and b-3 indicate classification of convex versus concave 3D images generated with different types of stimuli. RDS = random dot stereogram.

The high sensitivity of MVPA allows for the detection of subtle differences between conditions of interest and the comparison of neural patterns between cortical ROIs of the human brain. In this study, we performed MVPA for the ROIs in MATLAB. The linear support vector machine (SVM) implemented in MATLAB was used as the classifier for MVPA. Two main types of classification were performed. In the first classification type, SVM was trained and tested using activity patterns evoked by shapes with the same type of stimuli (e.g., using data from the shapes by RDS for both training and testing). This classification type was used to assess whether reliable neural activity patterns are induced by convex and concave shapes with a specific stimulus type. The second classification type, transfer convex versus concave classification, used activity patterns evoked by images with different types of stimuli for training and testing (e.g., using activity evoked by the shapes with RDS for training and the shapes with “lines with perspective” for testing). The main purpose of this second type of classification was to assess whether certain areas within the visual cortex exhibit common neuronal activity patterns in response to convex or concave shapes, irrespective of stimulus type. Figure 6 illustrates these classifications.

The MVPA procedure was conducted as follows: all fMRI time series were shifted two volumes (6 seconds) to account for the hemodynamic delay of the BOLD signal. For each ROI, voxels were selected from both the left and right hemispheres. These voxels were sorted from large to small response magnitude compared with the eye-fixation baseline condition. We selected the top 250 voxels from each ROI for classification. If the total number of voxels was less than 250 for a given ROI, the largest available number of voxels was used for the classification.

To estimate the response amplitude of each voxel of ROI within a stimulus block, we calculated the difference between the average BOLD signal of the first three volumes (after shifting by two volumes) after stimulus onset (denoted *avg1*) and the average BOLD signal value of the last two volumes (after shifting by two volumes) before stimulus onset (denoted *avg2*), or *avg1–avg2*. Finally, all difference values were transformed into *z* scores, and these *z* scores were used as the input for SVM training and testing.

To evaluate the performance of MVPA classification, the leave-one-run out crossclassification method was used to partition the EPI data into training and testing data sets consisting of different combinations of data from individual subject runs. For each individual subject ROI, the accuracies of all crossclassifications were averaged. Finally, the averaged accuracy of each individual ROI was averaged across all participants.

To assess whether the classification accuracy was reliable, permutation testing was performed to estimate the baseline of statistical significance. Classification was performed using randomly permutated fMRI patterns for all ROIs (i.e., the correspondences between the fMRI data and the class labels were randomized) and classification was performed in the same way as for normal nonpermutated data. We repeated this procedure 1,000 times to generate a distribution of classification accuracies. The 99.6th percentile (one-tailed, 12 ROIs) was used as the baseline for statistical significance.

## Results

### Behavioral Results

For the behavioral results for judging convex versus concave during the fMRI scan, the average accuracies of the runs after the exclusion of unqualified runs for participants ranged from 86.46% to 100% (mean ± standard deviation, 93.97% ± 6.54%). The analysis of variance (ANOVA) results showed that there were no significant difference between the average behavioral accuracies for the three types of stimuli (i.e., RDS, lines with perspective, lines with disparity): *F*(2, 16) = 1.364, *p* = .284. The detailed behavioral data are shown in Table 1 in the supplemental material.

### Classification of Convex Versus Concave 3D Images With the Same Type of Stimuli

To investigate whether the neurons in the ROIs are selective to convex or concave 3D images generated from RDS, black and white lines with perspective, and black and white lines with disparity, we measured the BOLD signals from the visual cortex and then fed the ROI data to MVPA using the same type of stimuli for training and testing (e.g., training using RDS data and testing using RDS data).

#### Classification of Convex Versus Concave Images Composed of Random Dots

**Figure 7.**
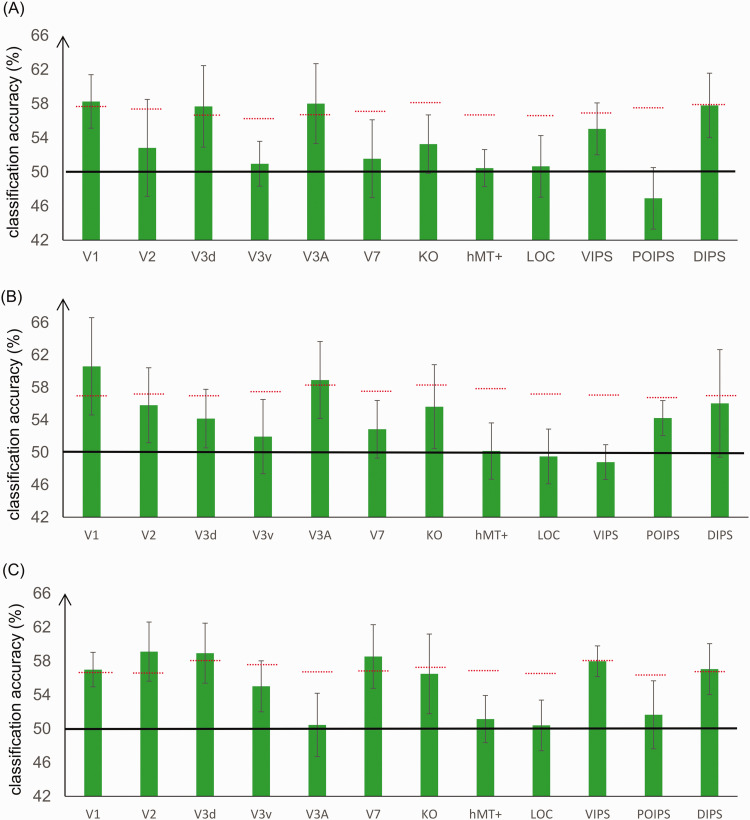
Classification accuracies for convex versus concave 3D images generated with the same type of stimuli. A: RDS data. B: Lines with perspective. C: Lines with disparity. The red horizontal dotted lines indicate the baseline of statistical significance for each ROI. The locations of these lines indicate the upper 99.6th percentile of the classification accuracy distribution for the permutated data. The black horizontal line indicates the chance level for the binary convex versus concave classification (50%). The error bars depict the standard error of the mean across subjects (*n* = 9). LOC = lateral occipital complex; hMT+ = the human middle temporal complex; KO = kinetic occipital area; VIPS = ventral intraparietal sulcus; POIPS = parieto-occipital intraparietal sulcus; DIPS = dorsal intraparietal sulcus.

This classification process is illustrated Figure 6a-1, and the results for all ROIs are shown in Figure 7A. Almost all ROIs showed classification accuracy (convex vs. concave) greater than chance for this binary classification (50%). Among these ROIs, V1, dorsal areas V3d, and V3A demonstrated classification accuracy higher than the baseline of statistical significance, and DIPS showed classification accuracy only slightly below the baseline of statistical significance.

#### Classification of Convex Versus Concave Images Composed of Lines With Perspective

This classification process is illustrated in Figure 6a-2, and the results for all ROIs are shown in Figure 7B. Again, many ROIs showed classification accuracy higher than chance (50%). Among these ROIs, V1 and dorsal area V3A demonstrated classification accuracy higher than the baseline of statistical significance.

#### Classification of Convex Versus Concave Images Composed of Lines With Disparity

This classification process is illustrated in Figure 6a-3, and the results for all ROIs are shown in Figure 7C. Many ROIs showed classification accuracy higher than chance (50%), and the ROIs for V1, V2, dorsal areas V3d and V7, and intraparietal area DIPS demonstrated classification accuracy higher than the baseline of statistical significance.

### Classification of Convex Versus Concave 3D Images With Different Types of Stimuli

In the second type of classification, SVM was trained and tested using different types of stimuli (different depth cues and visual elements) to investigate whether certain ROIs exhibit a common neural activity pattern in response to a given image (concave or convex), independent of the specific type of stimuli (e.g., training using images generated from black and white lines with perspective and testing using images generated from black and white lines with disparity).

#### RDS for Training and Lines With Perspective for Testing, and Vice Versa

**Figure 8.**
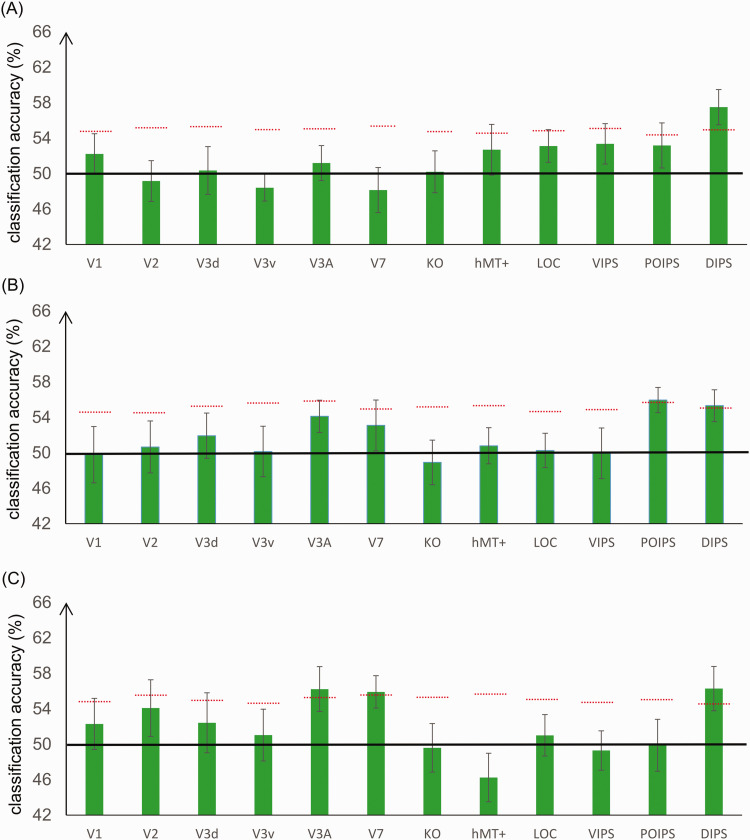
Classification accuracies for convex versus concave 3D images generated with different types of stimuli (different depth cues and elements). A: Random dot stereogram (RDS) and lines with perspective. B: Lines with perspective and lines with disparity. C: RDS and lines with disparity. The red horizontal dotted lines indicate the baseline of statistical significance for each ROI. The locations of these lines indicate the upper 99.6th percentile of the classification accuracy distribution for the permutated data. The black horizontal line indicates the chance level. The error bars depict the standard error of the mean across subjects (*n* = 9). LOC = lateral occipital complex; hMT+ = the human middle temporal complex; KO = kinetic occipital area; VIPS = ventral intraparietal sulcus; POIPS = parieto-occipital intraparietal sulcus; DIPS = dorsal intraparietal sulcus.

The classification process is illustrated in Figure 6b-1. In this classification task, we trained SVM using the 3D shapes generated by the RDS and tested SVM using shapes generated by black and white dotted lines with perspective, and vice versa. The results are shown in Figure 8A. Only intraparietal area DIPS showed classification accuracy higher than the baseline of statistical significance. Paired-sample *t* test revealed that the mean values of these two types of classification were not significantly different in all ROIs (false discovery rate <0.05), and more strictly, if the correction of multicomparison problem is not considered, only V2 showed a *p* value of less than .05 (*p* = .029). However, the classification accuracies in both directions were around chance level, which does not affect our conclusion; therefore, averaging the results of the two types for each ROI remains accurate.

#### Lines With Perspective for Training and Lines With Disparity for Testing, and Vice Versa

This classification is shown in Figure 6b-2. In this classification task, we trained the SVM using 3D shapes generated by black and white dotted lines with perspective and tested the SVM using shapes generated by black and white dotted lines with disparity (and vice versa). The results are shown in Figure 8B. Only the intraparietal areas POIPS and DIPS showed classification accuracy higher than the baseline of statistical significance. Paired-sample *t* test showed that the mean values of these two types of classification were not different significantly in all ROIs (false discovery rate <0.05), and more strictly, if correction of the multicomparison problem is not considered, only the VIPS showed a *p* value of less than .05 (*p* = .049). However, the classification accuracies in both directions were around chance level, which does not affect our conclusion; therefore, there is no problem that we averaged the results of the two types for each ROI.

#### RDS for Training and Lines With Disparity for Testing, and Vice Versa

This type of classification is illustrated in Figure 6b-3. We examined the classification accuracy for images generated using different visual elements but the same depth cue (RDS for training and lines with disparity for testing and vice versa). Paired-sample *t* test showed that the mean values of these two types of classification were not different significantly in all ROIs; therefore, we averaged the results of the two types for each ROI. The results are shown in Figure 8C. The dorsal areas V3A and V7, as well as IPS area DIPS, demonstrated a classification accuracy higher than the baseline of statistical significance.

The baselines of statistical significance require some explanation. The baselines in Figure 7 are generally higher than the baselines in Figure 8. Generally speaking, if the label-shuffling procedure could really randomize the data structure, the baselines should be determined by the numbers of repetitions, samples, and participants, irrespective of the type of classification and the baselines in Figures 7 and 8 should almost be same. But in our case, the baselines in Figure 8 are the averaged values of the classification results of transfer classification in two directions of the shuffled data, and the variance of averaged accuracy is decreased compared with every component’s classification accuracy; therefore, the baselines are lower in Figure 8.

## Discussion

In this study, we examined the processing of different visual elements (dots and lines) and depth cues (disparity and perspective) by different subregions of the visual cortex using fMRI and MVPA to elucidate the mechanisms of 3D image processing. To examine the processing of visual elements, measurements were conducted using similar images (a convex or concave shape) generated by an RDS and using black and white dotted lines, both with binocular disparity as the depth cue. The processing of depth cues was examined by measuring the responses to similar images generated by the same elements (black and white dotted lines) but with different depth cues (disparity or perspective).

The major findings can be summarized as follows: (a) When depth cue and element were the same, the regions with activity that showed significant classification accuracy by SVM were mainly located in the lower and mid-level visual areas, and the higher order DIPS region also demonstrated significant or near significant classification accuracy; (b) When the depth cues and elements were different, the DIPS region and other higher order areas demonstrated significant classification accuracy. These results suggest that, in general, lower level visual areas generate different activity patterns in response to the same image with different depth cues and elements, while higher level regions generate similar activity patterns in response to the same image with different depth cues and elements.

### Differential Responses of Various Low- and High-Level Visual Cortices to Depth Cues/Elements

It is believed that the visual system is split into two separate pathways after the primary visual cortex (V1): a dorsal pathway to the parietal cortex and a ventral pathway to the inferior temporal cortex. Visual information is processed progressively in a hierarchical manner from the lower to the higher order visual areas. Lower order visual areas in each pathway mainly process simple attributes, such as motion direction, orientation, and speed, whereas the visual attributes processed by the higher order visual areas of each pathway are much more complex, culminating in recognition of objects and complex shapes. We hypothesized that the lower order visual areas would show higher accuracy for the same cue and element types in the convex versus concave classification condition, and that some higher order visual areas would show substantial accuracy in both same-cue/element type and different-cue/element type (transfer) convex versus concave classification conditions. In general, the MVPA results are in line with our expectations.

(1) The classification accuracy of V1 was above the baseline of statistical significance for all convex versus concave classifications with the same cue and element type. This type of classification includes three subtypes depending on the stimuli used. In the same-cue/element type (lines with perspective) convex versus concave classification condition, the convex and concave shapes are distinguished by line orientation, and V1 neurons are highly sensitive to lines of different orientation (Hubel & Wiesel, 1959). For the same-cue and element-type (RDS or lines with disparity) convex versus concave classification, it is possible that the high accuracy was dependent on the selectivity of V1 neurons to the signs of disparity gradient: the disparity sign is different for concave and convex forms, and V1 neurons are highly sensitive to the sign of binocular disparity (Preston et al., 2008). In addition, the convex shapes provided information of uncrossed disparity and the concave shapes provided information of crossed disparity, which may differentially activate populations of V1 neurons. It is also possible that the high classification accuracy of V1 for these two conditions may have been aided by the difference in image depth (near or far to the fixation marker image plane).

(2) For results of same-type stimuli convex versus concave classifications in V2, the description is as follows:

Accuracy was slightly higher than the chance level (50%) for convex versus concave classifications with the RDS; accuracy was higher than chance level but lower than the baseline of statistical significance for convex versus concave classifications with lines with perspective; accuracy was higher than the baseline of statistical significance for convex versus concave classifications with lines with disparity. Previous studies have reported that V2 is related to process disparity (Preston et al., 2008; Thomas et al., 2002); therefore, it is reasonable that classification accuracy was higher than the baseline of statistical significance on the lines with disparity. However, the results of our study for the RDS stimuli differ from those in the previous study. This may have been caused by the fact that the single-cell study of Thomas et al. (2002) reported that 68 of a total 165 V2 neurons showed selectivity to the disparity stimuli used. As only some neurons show selectivity to disparity, it is possible that the activity of neurons that are not selective to disparity may affect the classification accuracy. In addition, the previous studies (Preston et al., 2008) mainly adopted planes in different depth positions. Because the local disparities on different parts of the convex/concave shape are different, while the disparities on different parts of the plane are same, it may have been harder for the SVM to classify convex versus concave than to classify depth positions on planes; therefore, the same-type stimuli classification accuracy for RDS (52.83%) was only slightly higher than chance level. For convex/concave shapes from lines with perspective, we can also partially view them as shapes from their texture. Previous studies have shown that V2 is selective to texture (Freeman et al., 2013; Ziemba et al., 2016); therefore, the classification accuracy for lines with perspective (55.80%) was higher than chance level but still did not reach the baseline of statistical significance (57.31%).

The classification accuracies for convex versus concave 3D images generated with different types of stimuli were not higher than the baseline of statistical significance, indicating that V2 is more involved in the lower attributes of the shapes.

(3) The classification accuracy of V3d was significant for the same cue (disparity) and element (dots or lines) convex versus concave condition, possibly because V3d neurons are sensitive to binocular disparity. In an fMRI study by Preston et al. (2008), neurons in V3d demonstrated selectivity to absolute disparity, while Chandrasekaran et al. (2006) reported that fMRI activity in area V3d is related to performance in binocular disparity-defined shape judgments. Therefore, the high classification accuracy in these two conditions is consistent with the response properties of V3d neurons. Conversely, the classification accuracy of V3d activity was below significance for the convex versus concave classification of images generated by lines with perspective, suggesting that V3d neurons are not selective to perspective or that perspective-selective neurons are not clustered densely enough (and thus the local signal is not strong enough) for SVM classification. The classification accuracy was also not significant for the transfer convex versus concave classification conditions using different depth cues. We speculate that this classification requires high-order processing (greater integration), while V3d is an early visual area in the dorsal pathway. At a lower level of processing, differences in training and testing conditions would induce different activity patterns for the same shape type, resulting in poor classification accuracy.

(4) The classification accuracy of the dorsal area V3A was significant for the same-cue and element convex versus concave conditions for the RDS and lines with perspective as well as for the transfer condition between RDS and lines with disparity. These findings may be explained by the selectively of V3A neurons for disparity (Goncalves et al., 2015). Moreover, our previous fMRI study showed that V3A is selective to stereoscopic convex–concave shapes (Li & Shigemasu, 2019). The disparity patterns between the convex and concave shapes defined by the RDS were different, so the high accuracy for the RDS convex versus concave classification condition is consistent with the known response properties of V3A neurons. In addition, there is evidence that basic perspective processing can be performed in V3A (Welchman et al., 2005), thereby accounting for the significant accuracy for lines with perspective convex versus concave classification. While both RDSs and lines with disparity create 3D convex and concave shapes by binocular disparity, the classification accuracy was significant for the RDS condition but was around chance level for the lines with the disparity condition. As V3A neurons are selective for shape, it is reasonable that the classification accuracy would be high for the RDS. Alternatively, the shapes generated by lines of disparity were depicted by conflicting cues: the disparity produced the convex and concave shapes, whereas the horizontal and vertical parallel lines created the flat plane. As mentioned earlier, V3A neurons are also involved in the processing of perspective; therefore, V3A’s accuracy in the lines with disparity condition was near chance level.

The V3A classification accuracy for the RDS/lines with disparity transfer condition was also significant, consistent with our previous MVPA study (Li & Shigemasu, 2019), in which we found that V3A neurons showed a common activity pattern to convex and concave shapes defined by disparity in two different orientations and depth positions. It is thus possible that area V3A is involved in a generalized representation of shape defined by binocular disparity and does not depend on element type (random dots vs. lines). It is worth mentioning that, although the shapes generated by lines of disparity were depicted by conflicting cues, the accuracy was still high. This may be due to the fact that the elements of RDS and lines with disparity are different: the RDS consists of random dots, and the lines with disparity consist of lines; as the elements are different, the conflict-cue effect may be weakened and the SVM must be more biased to disparity, resulting in high accuracy.

For transfer classification between lines with perspective and lines with disparity, the accuracy in V3A was lower than the baseline of statistical significance. There are two possible reasons for this result: the first possibility is that there are common patterns for the stimuli of lines with perspective and lines with disparity, but both types of stimuli contain line elements, and the conflicting cues contained in lines of disparity stimuli affect the transfer classification, therefore the accuracies were low in V3A; the other possibility is that there is no common pattern for each of the pairs of stimuli. Further study is required to distinguish which of these possibilities is correct.

(5) IPS regions: The classification accuracy of the DIPS was around the baseline of statistical significance for the same-cue and element condition and higher than the baseline of statistical significance for all transfer (different depth cue or element) conditions. Mid-order and higher order visual cortices appear to process first-order and second-order depth information (such as slant and curvature, respectively) from 3D object structures. The areas involved in processing higher order depth features of objects in the dorsal cortex include the hMT+, V3A, V7, and regions along the IPS (Alizadeh et al., 2018; Freud et al., 2016; Georgieva et al., 2009; Janssen et al., 2018; Katsuyama et al., 2011). Many previous studies have found that intraparietal areas are involved in 3D shape processing. For example, there is evidence that the CIP in monkeys, which corresponds to the VIPS, V7/IPS0, or V7A in humans (Konen et al., 2013; Orban, 2016), is involved in the representation of 3D curvature defined by disparity (Alizadeh et al., 2018). The CIP region may also be a locus where different types of depth cues are integrated, including disparity and linear perspective (Tsutsui et al., 2001). The CIP area projects to anterior regions of the intraparietal area (AIP) and may belong to a larger object-processing network (Erlikhman et al., 2018). There is also evidence that the AIP region in nonhuman primates is sensitive to 3D curvature and 3D shape (Joly et al., 2009; Srivastava et al., 2009). The AIP of nonhuman primates is believed to correspond partially to the human DIPS (Orban, 2016), and Durand et al. (2009) found that some human DIPS regions are sensitive to depth structure. Considering these findings, it is reasonable that accuracy was high in the DIPS for all types of classification. In addition, in the two-stream theory proposed by Goodale and Milner (1992), information in the dorsal stream is used for the unconscious control of action, such as the movement of the body guided by visual input. It is possible that the 3D information in the dorsal stream ultimately serves visually guided actions (Cohen & Andersen, 2002; Sakata et al., 1998). Accordingly, some regions in the posterior parietal cortex involved in motor planning respond to depth signals, including AIP for grasping (Srivastava et al., 2009; Theys, Srivastava, et al., 2012), the parietal reach region (Bhattacharyya et al., 2009), and the lateral intraparietal area (Durand et al., 2007). This information, independent of depth cue type and element type, may be required for rapid visually guided actions. As this information is more generalized, it does not depend on the specific nature of the depth cue and element. Therefore, the DIPS showed high classification accuracy for both same-cue/element type and transfer conditions.

In summary, the common pattern in the DIPS may be related to two functions. First, it is possibly related to more generalized shape presentation that does not depend on stimulus type. As the visual information goes along the visual stream, it is possible that information from different cues is fused into a single representation at a certain stage, and that this information then flows to the higher areas. Therefore, the DIPS will contain this generalized information. Second, it is possibly related to potential information used for action. Anatomically, part of the IPS area is connected to the ventral premotor cortex (Matelli et al., 1986), especially to motor area F5. Previous animal studies have demonstrated that the IPS projects to F5 (Theys, Pani, et al., 2012; Theys et al., 2013), which is related to specific object-related hand movements and presentation of a 3D object without subsequent manipulation (Murata et al., 1997). Action planning, such as grasping the convex or concave shapes, is related to the shape and not related to the cues defining the shape. The common neural activity in the IPS may be related to potential information which flows from the IPS to motor-related areas, and this information may correlate with the shape of objects, which does not depend on depth cues. Also, there may be information feedback from F5 to parts of the IPS areas, which can improve the classification accuracy in the IPS areas; however, to the best of our knowledge, there are no previous studies that support the hypothesis of feedback from the premotor cortex to IPS areas. Further investigation is required on this point.

### The Role of Eye Movement

As most of the high accuracies we obtained were on the ROIs in the dorsal stream, and this stream serves visually guided actions (Goodale & Milner, 1992), is it possible that these high accuracies were related to the eye movement pattern while the participants observed the convex/concave stimuli. To answer this question, we conducted an additional behavior experiment in a dark room. An EyeLink II eye-tracker (SR-Research Ltd., Mississauga, Ontario, Canada) was used to track the eye movement while the participants completed the same tasks as those for the fMRI experiment: 10 participants (6 males, 4 females) were recruited for this experiment. For the analysis, we calculated the fixation position difference between the stimulus block and fixation block for the left eye (denoted by *dl*) and right eye (denoted by *d*r) separately; then, the difference between the right and left eyes (delta = *dr* − *dl*) was calculated. A two (shape: convex and concave) by three (cue: RDS, lines with disparity, and lines with perspective) repeated-measures ANOVA was performed. The ANOVA results showed that there was no main effect of shape, *F*(1, 9) = 0.638, *p* = .445, and no main effect of cue, *F*(2, 18) = 2.394, *p* = .12; also, there was no interaction between shape and cue: *F*(2, 18) = 2.154, *p* = .145. From the eye movement data, there was no specific movement pattern associated with the convex/concave shape; therefore, we believe that the high accuracies were not caused by eye movements during the experiment. However, we cannot exclude the possibility that the high accuracy was caused by potential information related to the shapes, which can be used for action.

### The Relationship Between Behavioral Results and MVPA Results

We checked the Pearson’s correlation coefficient between behavioral accuracies and “same-type stimuli classification” accuracies in each area on the data for the RDS, lines with perspective, and lines with disparity, respectively. The results showed that there was no correlation between the behavioral accuracies and the classification accuracies of the fMRI data for all the ROIs. The detailed results are shown in Table 8 in the supplementary material. The possible reasons we could not find a relationship between the behavioral results and the fMRI results are as follows: one possibility is that there is indeed no relationship between the behavioral performance and the neural activity pattern; another possibility is the limitations of our method: (a) the participants recruited for the experiment could perform the task very easily, the behavioral performance was good, and the variation of performance was small; (b) the difficulty of the task was constant during the fMRI scan, therefore, we could not measure performance changes systematically.

## Limitations

**Figure 9.**
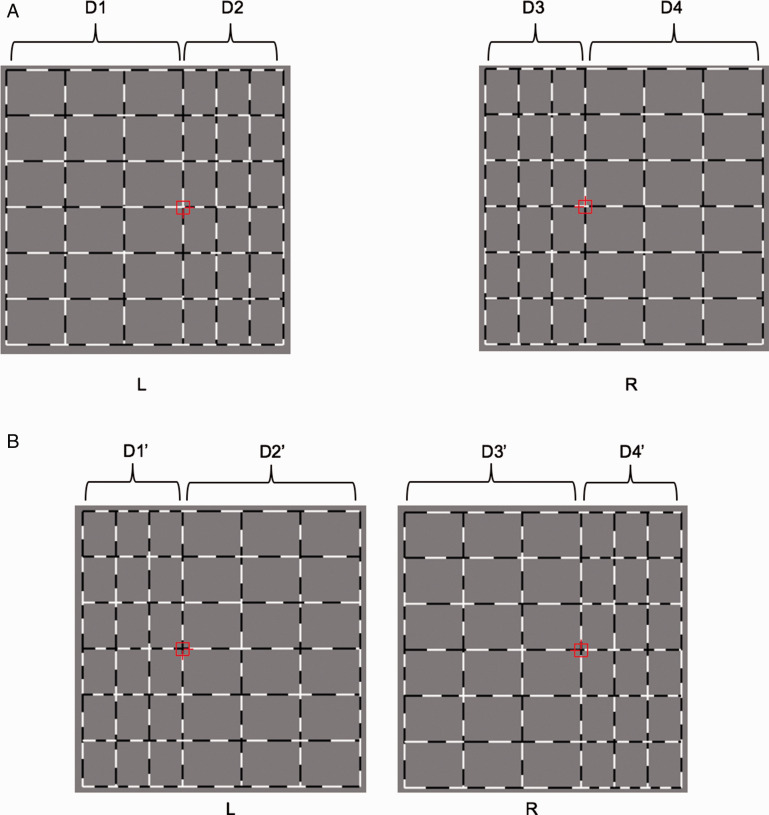
Illustration of monocular information for judging convex versus concave shapes. Shapes defined by black and white dotted lines with disparity are shown. L indicates the image projected to the left eye, and R indicates the image projected to the right eye. The stimuli shown in this figure are simplified for illustration and are not the real stimuli shown to the participants. A: Stimuli generating the convex shape. B: Stimuli generating the concave shape.

The first limitation of this study is the potential use of monocular information for concave versus convex classification. Monocular information may be used for two same-cue/element classification conditions (RDS and lines with disparity) as well as for one transfer classification with stimuli of RDS and lines with disparity. These monocular cues are explained for the lines with disparity in Figure 9 as the RDS condition is similar. Figure 9 shows the shapes defined by black and white dotted lines with disparity. For illustration, these images have greater disparity and fewer lines than those used in the experiments. Part A shows a stimulus depicting a convex shape, and Part B shows a stimulus depicting a concave shape. For the convex shape, the distance between the left side line and the fixation marker, D1 in (A) of Figure 9, is longer than the distance between the right side line and the fixation marker, D2 in (A) of Figure 9 (i.e., D1 > D2). For the concave shape, the distance between the left side line and the fixation marker, D1’ in (B) of Figure 9, is shorter than the length between the right side line and the fixation marker, D2’ in (B) of Figure 9 (i.e., D1′ < D2′). This difference between convex and concave stimuli may be used by the left eye for the lines with disparity convex versus concave classification. There is also monocular information accessible to the right eye for classification. For the convex shape, the left side is shorter than the right (i.e., D3 < D4), (A) in Figure 9, whereas, for the concave shape, the left side is longer than the right (i.e., D3′ > D4′), (B) in Figure 9. Similar differences can also be used for the RDS/lines with disparity transfer classification.

Although it is possible that this monocular information was used for classification, we think it is unlikely. First, in the real stimuli for experiments, the length difference between the left side and the right side relative to the fixation marker was only 5.05 mm, which is about 0.2°. Second and more importantly, there was no such monocular information for the black and white dotted lines with perspective; therefore, the transfer RDS/lines with perspective and the transfer lines with perspective/lines with disparity classifications could not be made using monocular cues. We conclude that there are common neural activity patterns in the DIPS region for the processing of shapes with different depth cues and elements.

The second limitation is that this study can only confirm that neurons in the DIPS contain common activity patterns for processing shapes with different depth cues and elements, but we cannot judge whether other areas for early visual areas contain this kind of common pattern. If we assume this case, both the higher and lower areas can have common neuron patterns irrelevant to stimulus elements or types; therefore, stimulus shapes can be decoded in both higher and lower areas for same-type stimuli classification. However, in the lower visual areas, the stimulus features (e.g., disparity or perspective) are also strongly represented; thus, they may be contaminated in classification analysis. Therefore, the transfer decoding performance may be degraded compared with higher regions. In addition, the MVPA method of fMRI also has its limitations: if we can obtain a significantly high accuracy in an area, we can conclude that this area contains information regarding the classes. In contrast, if we cannot obtain high classification accuracy in an area, we cannot conclude that this area does not contain information regarding the classes. This is because there are many factors that can affect the accuracy of classification; for example, the resolution of the fMRI is limited and each voxel may contain numerous neurons, and these neurons do not have to be activated uniformly. Therefore, it is possible to obtain accuracy at chance level even if the information of classes is included. We still believe, however, that a common pattern to a shape from different cues/elements exists in some middle or higher areas. The reasons for this are as follows: (a) From the two-stream theory, visual information is processed progressively from early to high visual areas, and early areas mainly process simple attributes, while visual attributes processed by higher order visual areas of each pathway are much more complex. (b) There is evidence that shows that some dorsal areas, such as the MT and V3B/KO, may be related to the integration of some depth cues (Armendariz et al., 2019; Ban et al., 2012). It is possible that information from different cues is fused into a single representation at certain stage, and that this information then flows to the higher areas. Therefore, the higher areas will contain this generalized information. This generalized information does not have to be generated in the high areas; it may be generated in some middle stages. (c) Some regions in the posterior parietal cortex that are involved in motor planning respond to depth signals (Bhattacharyya et al., 2009; Srivastava et al., 2009; Theys, Srivastava, et al., 2012), and information about 3D shapes is required. This shape information should not directly rely on depth cues and should be generalized. This generalized representation may be one of the reasons that we obtained relatively high accuracy in the DIPS.

The third limitation of this study is that the number of participants was small compared with some of the other fMRI studies that used the MVPA method. For example, data from 12 participants were used in the study of Murphy et al. (2013). However, there have also been studies in which a similar number of participants were recruited. In study of Preston et al. (2008), for example, eight participants were recruited. The reliability of results can be assured by a “permutation test,” and this method was used in many studies (Ban & Welchman, 2015). The baseline for statistical significance selected can ensure that if classification accuracy is higher than the baseline, it is rare that this accuracy is obtained by chance. Therefore, we believe that our results are valid.

## Conclusion

In this study, we demonstrate that lower order visual cortices process 3D shapes differently depending on the specific depth cues/elements. In contrast, high-order cortices such as the DIPS can process 3D shapes similarly regardless of the specific depth cue/elements.

## Supplemental Material

sj-pdf-1-ipe-10.1177_20416695211018222 - Supplemental material for Unique Neural Activity Patterns Among Lower Order Cortices and Shared Patterns Among Higher Order Cortices During Processing of Similar Shapes With Different Stimulus Types Click here for additional data file. Supplemental material, sj-pdf-1-ipe-10.1177_20416695211018222 for Unique Neural Activity Patterns Among Lower Order Cortices and Shared Patterns Among Higher Order Cortices During Processing of Similar Shapes With Different Stimulus Types by Zhen Li and Hiroaki Shigemasu in i-Perception
