# Supplementary material for: Unique Neural Activity Patterns Among Lower Order Cortices and Shared Patterns Among Higher Order Cortices During Processing of Similar Shapes With Different Stimulus Types
Source: Iperception. 2021 May 26;12(3):20416695211018222. doi: 10.1177/20416695211018222 (PMC8161881; doi:10.1177/20416695211018222)
Supplement: sj-pdf-1-ipe-10.1177_20416695211018222 - Supplemental material for Unique Neural Activity Patterns Among Lower Order Cortices and Shared Patterns Among Higher Order Cortices During Processing of Similar Shapes With Different Stimulus Types [file sj-pdf-1-ipe-10.1177_20416695211018222.pdf]

Table 1: Behavioral results of judging convex vs. concave during fMRI scan. For participant privacy, P1–P9 are used to denote their names.

	RDS	Lines with perspective	Lines with disparity	Average
P1	100.00	100.00	100.00	**100.00**
P2	100.00	100.00	100.00	**100.00**
P3	97.50	92.50	100.00	**96.67**
P4	100.00	95.83	100.00	**98.61**
P5	84.38	87.50	87.50	**86.46**
P6	100.00	95.00	97.50	**97.50**
P7	94.44	97.22	91.66	**94.44**
P8	81.25	81.25	81.25	**81.25**
P9	92.50	87.50	92.50	**90.83**
**Average**	**94.45**	**92.98**	**94.49**	**93.97**

Table 2: Classification accuracies for convex vs. concave 3-D images generated with RDS for all participants. “BaselineOfSig” is the baseline of statistical significance. For participant privacy, P1–P9 are used to denote their names.

	V1	V2	V3d	V3v	V3A	V7	KO	hMT+	LOC	VIPS	POIPS	DIPS
P1	67.86	78.57	85.71	50.00	82.14	57.14	39.29	50.00	53.57	67.86	32.14	53.57
P2	72.50	57.50	55.00	57.50	50.00	47.50	55.00	52.50	40.00	62.50	47.50	62.50
P3	57.50	45.00	60.00	47.50	47.50	42.50	55.00	42.50	50.00	50.00	50.00	52.50
P4	45.83	70.83	62.50	37.50	70.83	70.83	54.17	41.67	41.67	66.67	50.00	54.17
P5	62.50	65.63	68.75	65.63	71.88	37.50	75.00	53.13	46.88	56.25	68.75	65.63
P6	52.78	33.33	44.44	52.78	38.89	33.33	44.44	44.44	58.33	55.56	52.78	66.67
P7	47.22	33.33	58.33	52.78	58.33	63.89	58.33	61.11	36.11	47.22	41.67	33.33
P8	65.63	56.25	46.88	50.00	50.00	43.75	53.13	56.25	59.38	46.88	34.38	71.88
P9	52.50	35.00	37.50	45.00	52.50	67.50	45.00	52.50	70.00	42.50	45.00	60.00
**Average**	**58.26**	**52.83**	**57.68**	**50.96**	**58.01**	**51.55**	**53.26**	**50.46**	**50.66**	**55.05**	**46.91**	**57.80**
BaselineOfSig	57.64	57.72	56.88	56.83	56.99	57.34	58.46	57.25	57.01	57.29	58.04	58.06

Table 3: Classification accuracies for convex vs. concave 3-D images generated with lines with perspective. “BaselineOfSig” is the baseline of statistical significance. For participant privacy, P1–P9 are used to denote their names.

	V1	V2	V3d	V3v	V3A	V7	KO	hMT+	LOC	VIPS	POIPS	DIPS
P1	78.57	64.29	42.86	71.43	53.57	42.86	53.57	39.29	46.43	46.43	60.71	75.00
P2	82.50	60.00	60.00	35.00	67.50	65.00	85.00	35.00	47.50	55.00	50.00	15.00
P3	77.50	62.50	77.50	65.00	75.00	65.00	65.00	47.50	55.00	42.50	62.50	52.50
P4	29.17	37.50	54.17	41.67	58.33	41.67	50.00	58.33	33.33	54.17	58.33	87.50
P5	43.75	50.00	43.75	50.00	71.88	56.25	56.25	40.63	65.63	43.75	50.00	62.50
P6	66.67	55.56	44.44	41.67	61.11	66.67	69.44	66.67	38.89	38.89	58.33	50.00
P7	47.22	36.11	52.78	38.89	27.78	50.00	44.44	58.33	61.11	58.33	55.56	55.56
P8	62.50	81.25	59.38	68.75	50.00	40.63	34.38	53.13	50.00	50.00	50.00	56.25

P9	57.50	55.00	52.50	55.00	65.00	47.50	42.50	52.50	47.50	50.00	42.50	50.00
**Average**	**60.60**	**55.80**	**54.15**	**51.93**	**58.91**	**52.84**	**55.62**	**50.15**	**49.49**	**48.79**	**54.22**	**56.03**
BaselineOfSig	57.24	57.31	57.21	57.61	58.40	57.51	58.35	57.89	57.28	57.30	57.01	57.15

Table 4: Classification accuracies for convex vs. concave 3-D images generated with lines with disparity. “BaselineOfSig” is the baseline of statistical significance. For participant privacy, P1–P9 are used to denote their names.

	V1	V2	V3d	V3v	V3A	V7	KO	hMT+	LOC	VIPS	POIPS	DIPS
P1	60.71	64.29	39.29	46.43	46.43	50.00	57.14	46.43	46.43	53.57	42.86	64.29
P2	57.50	65.00	62.50	60.00	67.50	47.50	57.50	47.50	55.00	52.50	67.50	55.00
P3	50.00	55.00	62.50	55.00	47.50	55.00	40.00	57.50	52.50	57.50	35.00	52.50
P4	58.33	62.50	66.67	54.17	45.83	62.50	75.00	54.17	54.17	58.33	41.67	50.00
P5	59.38	37.50	65.63	68.75	28.13	56.25	81.25	62.50	40.63	68.75	59.38	68.75
P6	47.22	63.89	69.44	47.22	61.11	80.56	41.67	50.00	36.11	63.89	66.67	72.22
P7	52.78	75.00	55.56	47.22	55.56	55.56	44.44	33.33	61.11	52.78	41.67	50.00
P8	59.38	56.25	43.75	68.75	46.88	71.88	56.25	56.25	62.50	59.38	50.00	53.13
P9	67.50	52.50	65.00	47.50	55.00	47.50	55.00	52.50	45.00	55.00	60.00	47.50
**Average**	**56.98**	**59.10**	**58.93**	**55.00**	**50.44**	**58.53**	**56.47**	**51.13**	**50.38**	**57.97**	**51.64**	**57.04**
BaselineOfSig	56.88	56.62	58.19	57.66	57.40	56.89	57.27	57.54	57.08	58.15	56.38	57.01

Table 5: Results of transfer convex vs. concave classification between data of RDS and lines with perspective. “BaselineOfSig” is the baseline of statistical significance. For participant privacy, P1–P9 are used to denote their names.

	V1	V2	V3d	V3v	V3A	V7	KO	hMT+	LOC	VIPS	POIPS	DIPS
P1	53.57	46.43	64.29	42.86	55.36	62.50	39.29	66.07	58.93	57.14	57.14	64.29
P2	43.75	53.75	46.25	45.00	50.00	46.25	47.50	58.75	55.00	48.75	42.50	56.25
P3	52.50	38.75	58.75	57.50	58.75	52.50	56.25	46.25	45.00	65.00	61.25	61.25
P4	50.00	43.75	47.92	52.08	37.50	39.58	47.92	56.25	56.25	60.42	41.67	62.50
P5	54.69	53.13	54.69	46.88	54.69	56.25	48.44	50.00	46.88	51.56	56.25	43.75
P6	43.06	45.83	48.61	44.44	52.78	40.28	47.22	40.28	48.61	44.44	58.33	56.94
P7	51.39	51.39	51.39	50.00	50.00	43.06	48.61	47.22	51.39	56.94	45.83	55.56
P8	54.69	46.88	37.50	46.88	51.56	45.31	51.56	46.88	54.69	48.44	59.38	59.38
P9	66.25	62.50	43.75	50.00	50.00	47.50	65.00	62.50	61.25	47.50	56.25	57.50
**Average**	**52.21**	**49.16**	**50.35**	**48.40**	**51.18**	**48.14**	**50.20**	**52.69**	**53.11**	**53.36**	**53.18**	**57.49**
BaselineOfSig	54.97	55.37	55.41	55.06	55.21	55.52	54.93	54.62	55.35	55.17	54.83	55.29

Table 6: Results of transfer convex vs. concave classification between data of lines with perspective and lines with disparity. “BaselineOfSig” is the baseline of statistical significance. For participant privacy, P1–P9 are used to denote their names.

	V1	V2	V3d	V3v	V3A	V7	KO	hMT+	LOC	VIPS	POIPS	DIPS
P1	42.86	35.71	41.07	37.50	51.79	62.50	44.64	57.14	53.57	51.79	58.93	51.79

P2	41.25	61.25	51.25	46.25	52.50	56.25	36.25	48.75	51.25	53.75	52.50	53.75
P3	43.75	52.50	60.00	53.75	51.25	48.75	52.50	52.50	51.25	62.50	48.75	57.50
P4	41.67	60.42	62.50	54.17	47.92	37.50	45.83	43.75	54.17	31.25	54.17	47.92
P5	68.75	54.69	42.19	56.25	64.06	60.94	40.63	46.88	46.88	48.44	60.94	59.38
P6	59.72	47.22	58.33	54.17	54.17	59.72	58.33	56.94	43.06	54.17	55.56	66.67
P7	44.44	49.44	53.66	58.15	55.92	45.41	50.81	56.11	46.55	46.39	54.83	52.78
P8	53.13	42.19	51.56	39.06	51.56	57.81	53.13	51.56	53.13	53.13	54.69	53.13
P9	52.50	57.50	46.25	46.25	51.25	51.25	57.50	41.25	58.75	50.00	62.50	55.00
**Average**	**49.79**	**51.21**	**51.87**	**49.51**	**53.38**	**53.35**	**48.85**	**50.54**	**50.95**	**50.16**	**55.87**	**55.32**
BaselineOfSig	54.71	55.33	55.53	55.12	55.41	55.59	55.39	55.32	55.63	55.19	55.76	55.28

Table 7: Results of transfer convex vs. concave classification between data of RDS and lines with disparity. "BaselineOfSig" is the baseline of statistical significance. For participant privacy, P1–P9 are used to denote their names.

	V1	V2	V3d	V3v	V3A	V7	KO	hMT+	LOC	VIPS	POIPS	DIPS
P1	57.14	66.07	66.07	67.86	58.93	53.57	50.00	53.57	46.43	55.36	58.93	66.07
P2	61.25	57.50	37.50	42.50	58.75	51.25	58.75	60.00	61.25	52.50	57.50	62.50
P3	43.75	51.25	58.75	47.50	57.50	48.75	56.25	48.75	52.50	61.25	48.75	45.00
P4	52.08	66.67	52.08	54.17	60.42	62.50	60.42	43.75	58.33	45.83	45.83	52.08
P5	46.88	46.88	46.88	43.75	40.63	51.56	48.44	34.38	46.88	42.19	40.63	46.88
P6	48.61	58.33	54.17	50.00	54.17	56.94	36.11	51.39	58.33	44.44	52.78	52.78
P7	55.56	40.28	62.50	50.00	68.06	54.17	44.44	45.83	43.06	51.39	54.17	58.33
P8	39.06	42.19	37.50	42.19	50.00	59.38	40.63	35.94	42.19	40.63	32.81	64.06
P9	66.25	57.50	56.25	61.25	57.50	65.00	51.25	42.50	50.00	50.00	57.50	58.75
**Average**	**52.29**	**54.07**	**52.41**	**51.02**	**56.22**	**55.90**	**49.59**	**46.23**	**51.00**	**49.29**	**49.88**	**56.27**
BaselineOfSig	54.93	55.80	55.10	54.91	55.54	55.81	55.39	55.78	55.29	55.13	55.14	54.90

Table 8: Pearson's correlation coefficient between behavioral results and MVPA results. RDS, lines with perspective, and lines with disparity indicate the results between behavioral results and classification accuracies for convex vs. concave 3-D images generated with RDS, lines with perspective, and lines with disparity, respectively.

RDS

ROI	V1	V2	V3d	V3v	V3A	V7	KO	hMT+	LOC	VIPS	POIPS	DIPS
Pearson Correlation	-0.182	-0.003	0.208	-0.371	-0.001	0.255	-0.521	-0.548	-0.268	0.543	-0.113	-0.39
Sig. (2-tailed)	0.64	0.995	0.59	0.325	0.998	0.508	0.15	0.127	0.485	0.131	0.772	0.299

Lines with perspective

ROI	V1	V2	V3d	V3v	V3A	V7	KO	hMT+	LOC	VIPS	POIPS	DIPS
Pearson Correlation	0.244	-0.441	-0.126	-0.422	-0.157	0.27	0.592	-0.161	-0.271	0.221	0.526	-0.081
Sig. (2-tailed)	0.526	0.235	0.746	0.257	0.687	0.482	0.093	0.68	0.481	0.567	0.146	0.835

Lines with disparity

ROI	V1	V2	V3d	V3v	V3A	V7	KO	hMT+	LOC	VIPS	POIPS	DIPS
Pearson Correlation	-0.285	0.402	0.273	-0.59	0.398	-0.277	-0.206	-0.214	-0.244	-0.374	-0.128	0.04
Sig. (2-tailed)	0.457	0.284	0.478	0.095	0.289	0.471	0.596	0.579	0.527	0.322	0.744	0.919
